# Supplementary material for: Super-Earths, M Dwarfs, and Photosynthetic Organisms: Habitability in the Lab
Source: Life (Basel). 2020 Dec 24;11(1):10. doi: 10.3390/life11010010 (PMC7823553; doi:10.3390/life11010010)
Supplement: Supplementary file 1 [file life-11-00010-s001.pdf]

life

Article

Super-Earths, MDwarfs, and Photosynthetic Organisms: Habitability in the Lab

Riccardo Claudi [1,*], Eleonora Alei [1,2], Mariano Battistuzzi [3,4], Lorenzo Cocola [5], Marco Sergio Erculiani [6], Anna Caterina Pozzer [1,3], Bernardo Salasnich [1], Diana Simionato [3], Vito Squicciarini [1,7], Luca Poletto [5] and Nicoletta La Rocca [3,4]

1 Osservatorio Astronomico di Padova, INAF, 35122 Padova, Italy; Elalei@phys.ethz.ch (E.A.); caterina.pozzer@inaf.it (A.C.P.); bernardo.salasnich@inaf.it (B.S.); vito.squicciarini@inaf.it (V.S.)
2 Institute for Particle Physics and Astrophysics, ETH Zurich, 8093 Zurich, Switzerland
3 Department of Biology, University of Padova, 35131 Padova, Italy; mariano.battistuzzi@phd.unipd.it (M.B.); diana.simionato@unipd.it (D.S.); nicoletta.larocca@unipd.it (N.L.R.)
4 Centro di Ateneo di Studi e Attività Spaziali (CISAS) Giuseppe Colombo, 35131 Padova, Italy
5 Institute for Photonics and Nanotechnologies, CNR, 35131 Padova, Italy; lorenzo.cocola@cnr.it (L.C.); luca.poletto@cnr.it (L.P.)
6 Istituto di Radioastronomia, INAF, 40129 Bologna, Italy; marco.erculiani@inaf.it
7 Department of Physics and Astronomy, University of Padova, 35121 Padova, Italy
* Correspondence: riccardo.claudi@inaf.it

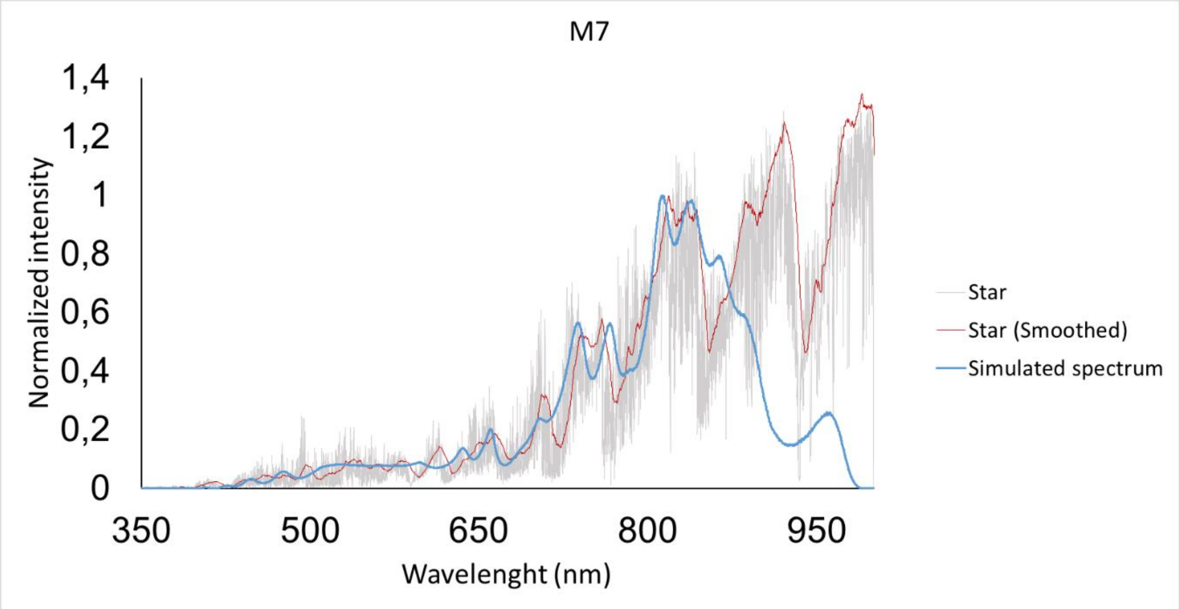

Figure S1. M7 V star input spectrum as it appears before (in light gray), and after (in red) the smoothing process. The emitted spectrum (in blue) is superimposed. The smoothing process reduces the resolution of the input spectra. Hence, the stellar simulator is able to better reproduce the input spectrum following the depletion of flux due to large atomic absorptions or molecular bands.

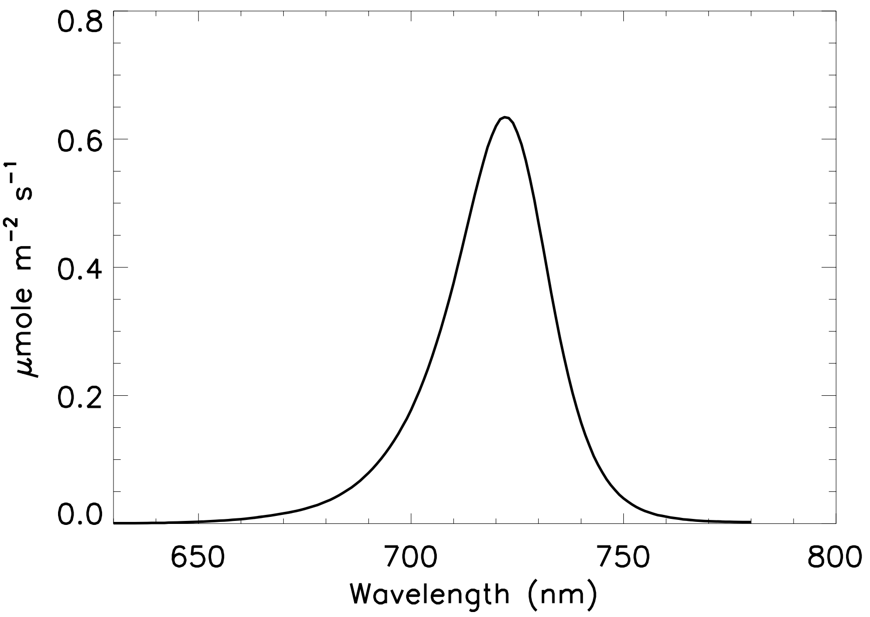

Figure S2. Emitted FR light measured by the FLAME VIS-NIR spectrograph. The central wavelength is 720\ nm and the full width at zero level is 130 nm with a wavelength range 650 nm and 780 nm. The luminosity of this lamp is 2.3 μmole m^{-2} s^{-1} in the PAR and 20μmole m^{-2} s^{-1} in the total working range.

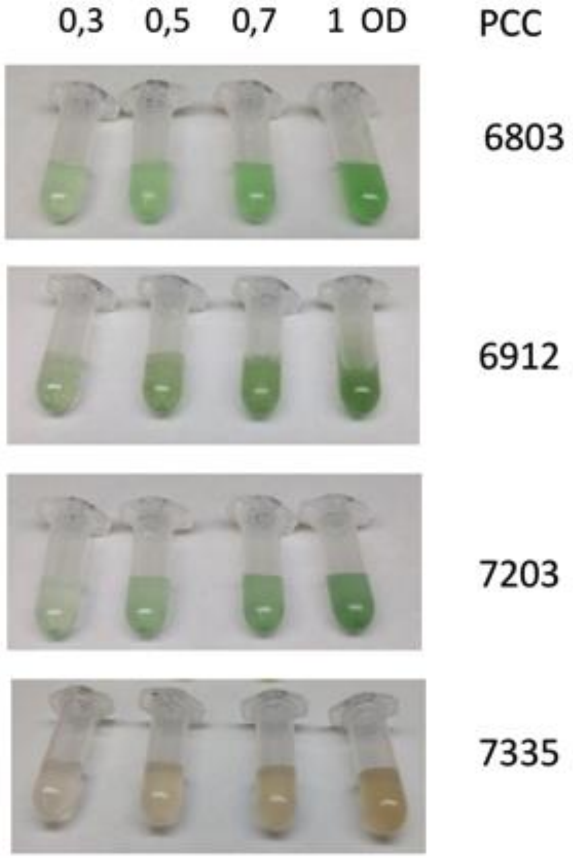

Figure S3. Different cultures of the selected cyanobacteria with different optical density, before the 20 μl spots were deposited on the Petri's plate.

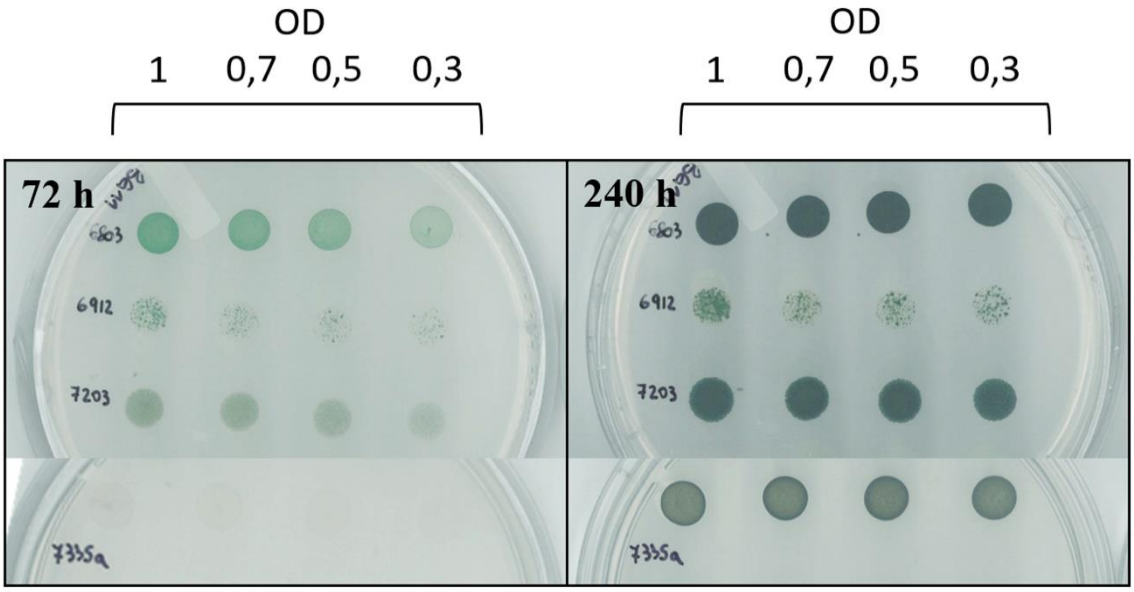

Figure S4. Examples of a BG-11 agar plate with S. sp. PCC6803, *C. fritschii* PCC6912 and *C. thermalis* PCC 7203 spots and a BG-11-ASN III agar plate with S. sp. PCC7335. Plates are shown after 72 and 240 h of exposure under the M-dwarf simulated spectrum.

Table S1. Averaged values (n=6) of the F_0 incremental ratio obtained for several organisms under different light sources. The considered error is 1 σ.

Light Source	PCC			
	6803	6912	7203	7335
SOL	11.88±2.92	2.00±0.56	9.24±1.82	2.27±0.22
M7	13.37±4.20	1.82±0.41	9.27±2.69	2.62±0.31
FR	0.35±0.13	0.63±0.26	0.78±0.26	1.08±0.08
